# Cellulose Ester-Based Aerogel: Lightweight and Highly Water-Absorbent

**DOI:** 10.1021/acsomega.3c07658

**Published:** 2024-01-09

**Authors:** Kadir Aksu, Mehmet Kaya

**Affiliations:** † Department of Chemistry, Faculty of Arts and Sciences, Ordu University, Ordu 52200, Türkiye; ‡ Department of Chemistry, Faculty of Arts and Sciences, Recep Tayyip Erdoğan University, Rize 53100, Türkiye

## Abstract

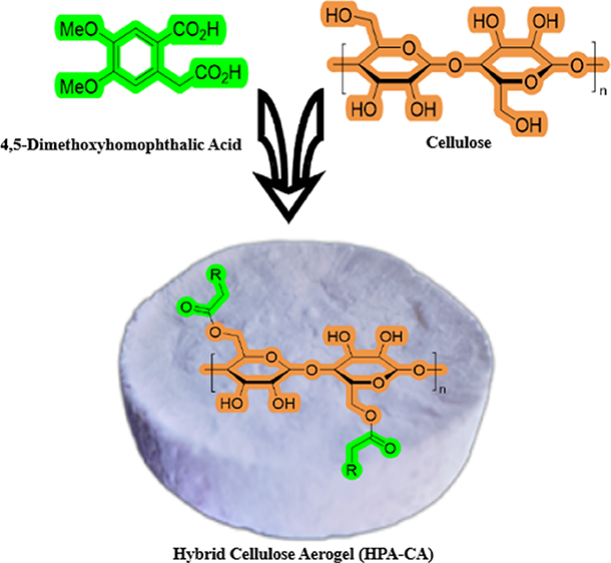

Cellulose was extracted from waste generated by pruning tea stem wastes. The interaction between pure cellulose and homophthalic acid produced a light (0.22 g·cm^–3^) and eco-friendly hybrid aerogel product that is highly absorbent (85 g of water per 1 g of aerogel). The product has a Brunauer–Emmett–Teller surface area of 221 m^2^·g^–1^. In addition, the product was analyzed for its structural and functional properties using scanning electron microscopy, Fourier transform infrared, and X-ray diffraction. The methodology employed in this study is uncomplicated, utilizing easily accessible and sustainable biowaste at a low cost. As a result, the current process is well-adapted for industrial-scale production, with the potential for significant advancements in the field of green materials.

## 1 Introduction

In the modern world, significant amounts of agricultural crop residues and wastes are produced at varying levels of productivity. These wastes can sometimes be evaluated in uneconomic or ecologically dangerous ways. In such cases, concerns such as increased costs and pollution may arise due to the increasing need for energy and raw materials. Present-day necessities are propelling scientists to explore sustainable alternatives that can substitute nonrenewable resources in the material realm.^1−6^ In recent times, agricultural waste has been repurposed into different industrial products. Cellulose, an abundant biopolymer on Earth, is often used as a raw material in these products as it is a renewable, natural, organic, biocompatible, and biodegradable resource.^7−10^ The emergence of cellulose as an alternative to conventional materials has gained attention due to its potential benefits.

One of the products produced recently from cellulose are cellulose aerogels. Cellulose aerogels are a viable cellulose-based alternative product with promising potential for diverse applications.^11−15^ These aerogels are among the lightest materials known, exhibiting an extremely porous structure due to the air volume that they contain, about 90–99%.^16^ Researchers have expressed significant interest in aerogels with advanced properties and a three-dimensional cross-linked network structure, primarily due to their high surface area, low density, and exceptional thermal insulation capabilities.^17^ In recent years, there has been a growing amount of attention being paid to cellulose-based aerogels, thanks to their flexibility, increased strength, biocompatibility, and biodegradability. While aerogels made with silica as their main component are generally produced using the sol–gel method, this technique is not appropriate for cellulose-based aerogels due to the scaffold structures of precipitated porous cellulose collapsing rapidly.^18^ Consequently, cellulose aerogels have been created through the use of the freeze-drying method in combination with specific chemical agents for cross-linking. Cellulose aerogels are created via physical or chemical cross-linking of a cellulose solution, utilizing the plentiful hydroxyl groups in cellulose that form hydrogen bonds or undergo chemical esterification to establish linked networks.^19^ Cross-linking is a popular methodology to ameliorate the structural properties and strength of polymers for diverse applications. Urea derivatives and multifunctional carboxylic acids are frequently employed cross-linkers for cellulosic materials.^20^ This selection guarantees the conservation of aerogel’s biodegradability and eco-friendliness, in addition to supporting a manufacturing process that is environmentally sustainable.

In this research, we describe the production and analysis of cellulose aerogel cross-linked with a highly lightweight, environmentally friendly, and biocompatible homophthalic acid (HPA) derivative that we synthesized in a previous study. The interest of cellulose in carboxylic acid-derived compounds and the positive effect of these acids on the properties of cellulose led us to conduct new research on the carboxylic acid–cellulose interaction in this study. In addition, the easy, cheap, and high-yield synthesis of HPA-derived compounds, their nontoxic effects, and a wide range of biological activity potentials played an active role in the selection of HPA.^21^ The cellulose that we employed in this study was derived from tea stem pruning waste, a byproduct of substantial volumes produced annually by tea plantations. In this study, the production process utilized HPA as a natural cross-linker, known for its environmentally friendly, nontoxic, and biodegradable properties, and for its ability to provide excellent swelling properties, to cellulose. Advanced measurement techniques were employed to investigate the structural and morphological properties, as well as the superabsorbent structure and flexibility of the cellulose aerogel, which was found to have superior properties. Advanced measurement techniques were used to investigate the structural and morphological properties of cellulose aerogel, which was found to have superior properties, as well as its superabsorbent structure and flexibility. Scanning electron microscopy (SEM), Fourier transform infrared (FTIR) spectroscopy, X-ray diffraction (XRD), and Brunauer–Emmett–Teller (BET) surface area were used for detailed analysis of the product. Although there are some studies in the literature on cellulose esterification and cellulose aerogel production, aerogel production from cellulose obtained by recycling local waste is not very common. In addition, there are limited studies in the context of “green chemistry” on the production and detailed characterization of carboxylic acid-modified hybrid aerogels that provide functionality and superior properties^22−26^ such as HPA. We believe that this research will provide important information about the potential uses and production techniques of biopolymers and green industrial aerogels, thus adding a significant value to the academic field. We are of the opinion that this research will offer significant knowledge on potential uses and manufacturing techniques of biopolymers and green industrial aerogels, thereby adding a meaningful value to the academic sphere.

## 2 Experimental Section

### 2.1 Characterization

For the SEM study, aerogel bulk samples on carbon tape were covered with a thin layer of gold before being placed in the SEM chamber. FTIR spectroscopy was conducted in the range of 3500–600 cm^–1^ using a Spectrum-100 FTIR spectrometer with a 4 cm^–1^ resolution. The XRD pattern for the products were produced using a Rigaku DMAX-3C automated diffractometer, with Ni-filtered Cu *K*_β_ radiation (40 kV and 30 mA). Diffractograms were obtained from 5 to 40° with a 3°/min scan rate. Nitrogen adsorption/desorption isotherms were obtained using an Autosorb-iQ-2 analyzer from Quantachrome Instruments based in Florida, USA, within the relative pressure range of 0.05 < *P*/*P*_0_ < 1.00. Before conducting textural measurements at 77 K, the samples were pretreated by degassing at 100 °C for 5 h. Gas (helium) pycnometer (Quantachrome ULTRAPYC-1200e) was used to carry out skeletal density studies. The bulk density was also assessed through mercury intrusion porosimetry to confirm and verify the actual density of the aerogels.

### 2.2 Materials

All chemicals were of analytical grade and were acquired from Merck. An ecologically friendly process was used to extract and bleach cellulose fibers from pruned tea stem wastes (TSW). TSW sawdust is predominantly composed of cellulose, lignin, and hemicellulose, which were quantified using Wise’s chlorite method.^27^

### 2.3 Cellulose Production from Biowaste

The pruned plant material was washed extensively with deionized water and filtered using a cloth strainer to remove impurities, such as water-soluble sand and soil. A 5.0 g sample of the material was treated with an alkaline solution of 4% NaOH (250 mL) at 80 °C for three cycles. The resulting alkaline-treated fibers (2.0 g) underwent bleaching with either NaClO_2_ (0.3 g) or H_2_O_2_ (5.0 mL, 30% w/w) in a 1.7% acetic acid buffer (250 mL) at 80 °C for four cycles. The fibers were filtered and rinsed with deionized water through a cloth strainer until a neutral pH was achieved. The cellulose samples were subsequently oven-dried at 105 °C.^28^ To obtain microcrystalline cellulose (MCC), 25 mL of 2.5 N HCl solution was added to 1 g of cellulose sample and mixed for 1 h at 100 °C. The resulting MCC samples were then filtered through filter paper and washed with cold water.

### 2.4 Synthesis of HPA

In a 250 mL reaction flask, 10.00 g (48.03 mmol) of 3-(3,4-dimethoxyphenyl) cinnamic acid (**1**) was dissolved in 100 mL of THF. Then, 10% Pd/C (0.50 g, 4.7 mmol) was added, and a flask filled with hydrogen was fitted. The air in the reaction flask was removed under vacuum. The reaction mixture was stirred at room temperature under a hydrogen atmosphere for 24 h. The mixture underwent filtration, followed by removal of the solvent using an evaporator. 3-(3,4-Dimethoxyphenyl) propanoic acid (**2**) was obtained as a white solid in a yield of 99% (9.98 g, 47.47 mmol). Its structure was characterized by ^1^H NMR and melting point analysis. The obtained results were found to be consistent with the literature data.^29^

**Scheme 1:**
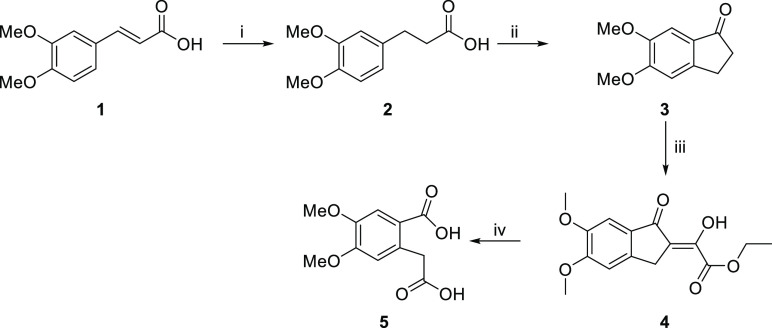
Synthesis of 4,5-Dimethoxyhomophtalic Acid (**5**): (i) H_2_/Pd–C, THF, rt, 24 h, 99%; (ii) H_3_PO_4_, P_2_O_5_, 80 °C, 1.5 h, 88%; (iii) NaOMe, Diethyl Oxalate, MeOH, 0 °C → rt, 18 h; (iv) KOH, H_2_O_2_, MeOH, rt, 16 h, 70%

A 26.13 g (266.68 mmol) portion of orthophosphoric acid was placed in a 250 mL beaker, followed by the addition of 46.86 g (330.16 mmol) of P_2_O_5_. The mixture was stirred at 80 °C until a clear solution formed. Then, 9.60 g (45.67 mmol) of 3-(3,4-dimethoxyphenyl) propanoic acid (**2**) was added to the prepared polyphosphoric acid and stirred at 400 rpm for 1.5 h. At the end of the reaction, the mixture was poured into 200 g of ice, neutralized with Na_2_CO_3_ and extracted with ethyl acetate (3 × 100 mL). The combined organic phases were dried over Na_2_SO_4_, and the solvent was removed under vacuum. The crude product was crystallized from 1:3 ethyl acetate/hexane. 5,6-Dimethoxy-1-indanone (**3**) was obtained in 88% yield (7.73 g, 40.22 mmol, yellow crystals). The spectral data is confirmed by the literature.^30^

A 10 mL portion of methanol was added to a 100 mL flask and placed in an ice bath. Sodium methoxide (NaOMe) was obtained by slowly adding 1.26 g (54.63 mmol) of sodium metal. Once prepared, NaOMe was added to 20 mL of toluene. In a separate solution, 5.00 g (26.01 mmol) of 5,6-dimethoxy-1-indanone (**3**) and 6.08 g (41.62 mmol) of diethyl oxalate were dissolved in 50 mL of toluene. This solution was then added dropwise to the prepared NaOMe solution over a period of 45 min at 0 °C. During the addition process, the temperature of the reaction mixture was carefully controlled to prevent it from rising above 0 °C. Following the addition, the mixture was stirred at room temperature for a duration of 18 h. After completing the reaction, the product (**4**) was not isolated before proceeding to the next step. The product (**4**) was suspended in 20 mL of MeOH and treated with solid KOH (8.75 g, 156.01 mmol) for 45 min. The reactant was added gradually while maintaining the reaction temperature below 50 °C. Once the addition concluded, the reaction mixture was stirred at room temperature for 1 h. Over a period of 3 h, 26.6 mL (29.50 g, 260.18 mmol) of a 30% H_2_O_2_ solution was slowly introduced to the mixture while making sure that the temperature stays below 64 °C using a cold-water bath. The reaction mixture was stirred at room temperature for 16 h and filtered, the solvent was removed with an evaporator, the residue was washed with ether, and the organic phase was discarded. The water phase was acidified with concentrated HCl until the pH 2–3 and then extracted with EtOAc (3 × 100 mL). The organic phases were combined and washed with brine before being dried over Na_2_SO_4_. The white crystal structure of 4,5-dimethoxyhomophtalic acid (**5**) called HPA was synthesized and yielded 70% (4.40 g, 18.32 mmol). The obtained spectral data are consistent with literature reports.^31^

### 2.5 Synthesis of HPA-MCC Hybrid Molecule (HPA-CA)

A saturated aqueous solution of HPA (4,5-dimethoxyhomophthalic acid) (**5**) was prepared by dissolving 3.00 g (12.49 mmol) of 4,5-dimethoxyhomophthalic acid (**5**) in water by heating to approximately 80–90 °C. For the modification of organic acid MCC, 675 mg (4.16 mmol cellulose monomer unit) was soaked overnight at room temperature in a saturated aqueous solution of 4,5-dimethoxyhomophthalic acid. The reaction mixture was placed in a 500 mL round-bottom flask and allowed to reflux at 120 °C for 3 h with magnetic stirring. The reaction mixture was diluted 1:1 range with deionized water, and the modified MCC was isolated by 20 times of repeated centrifugation at 4000 rpm for 10 min, replacing the initially clear supernatant with an equal volume of deionized water. The method for the synthesis of the 4,5-dimethoxyhomophthalic acid-MCC hybrid molecule (**7**) called HPA-CA was derived from the organic acid-modified CNC synthesis method described in the literature by Spinella et al.^32^

**Scheme 2:**
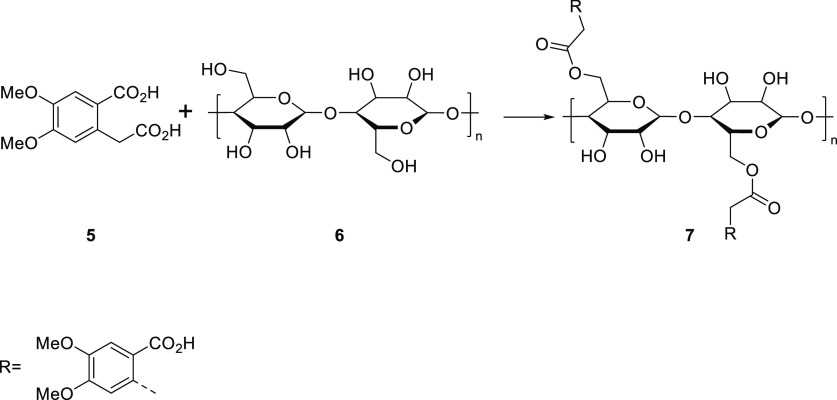
Synthesis of HPA-MCC Hybrid Molecule (HPA-CA)

### 2.6 Fabrication of Hybrid Aerogel (HPA-CA)

**Figure 1:**
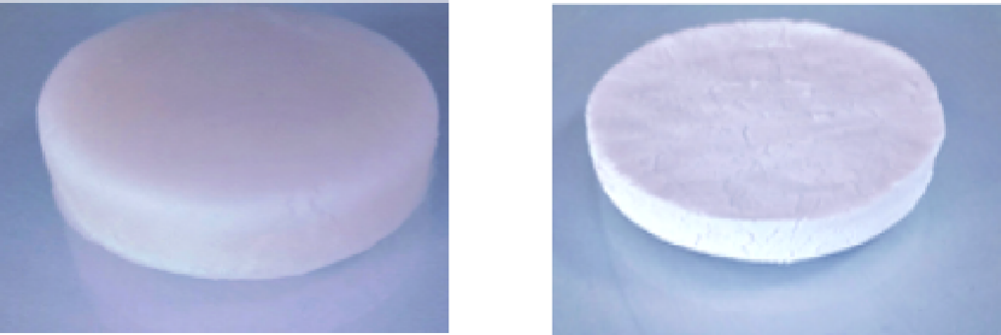
Photographs of the gel (left) and aerogel (right).

To create an aerogel from HPA and cellulose materials, the two substances were cross-linked. The HPA molecule retains a carboxylic acid group, which forms a linkage between two cellulose chains, inhibiting an alkaline environment or undergoing transesterification to repair a broken ester bond. To prepare cross-linked homophthalic acid-cellulose hybrid aerogel (HPA-CA), the HPA-CA material was dissolved in a tetrahydrofuran (THF) solvent at a weight ratio of 3%. The aerogel was fabricated by regenerating an HPA-CA THF solution in ethanol using the following procedure: The solution was poured onto a glass dish to form a 3 mm thick layer, which was then submerged in ethanol for regeneration-gelation. Subsequently, a sol–gel transition was induced via the solvent exchange mechanisms. The gel was subsequently rinsed with ethanol several times to eliminate THF. To reduce the risk of gel cracking and structural disturbance, it was precooled in a refrigerator overnight at +4 °C. The wet gel was freeze-dried, resulting in the generation of HPA-CA aerogel. Photographs of the gel (left) and aerogel (right) are depicted in Figure 1.

## 3 Results and Discussion

The study was initiated by the clear and scalable results yielded through the traditional isolation process of cellulose and the synthesis of HPA-derived aromatic carboxylic acid compounds as well as their facile and powerful interaction. The phenomenon of “green chemistry” and the biological activity of aromatic carboxylic acids across a wide range of fields provided further inspiration. Consequently, a more environmentally friendly and cost-effective avenue of research emerged.

### 3.1 TSW Component Analysis

Cellulose, one of the fundamental components of wood and one of the most abundant naturally occurring polymers composed of glucose, has garnered significant interest in the production of innovative aerogel materials due to its widespread availability and cost-effectiveness. Beyond applications in thin films, sponges, and fibers, cellulose offers the potential for creating high-value functional materials including cellulose nanocrystals, nanofibers, hydrogels, and aerogels. In the context of this study, the cellulose yield from TSW, as obtained in our previous work,^33^ plays a significant role. Table 1 presents essential data related to the content of extractives, cellulose, hemicellulose, and lignin in TSW. The TSW fibers were found to contain 31.58 wt % cellulose, 28.92 wt % hemicellulose, and 39.50 wt % lignin.

**Table 1:** Components of TSW

**sample**	**cellulose (%)**	**lignin (%)**	**hemicellulose (%)**
**TSW**	31.58	39.50	28.92

### 3.2 SEM Analysis

**Figure 2:**
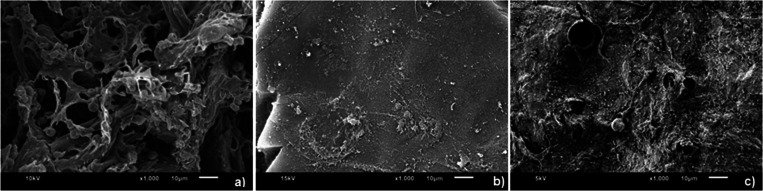
SEM images (×1000) of pure cellulose (a), MCC (b), and HPA-CA (c).

SEM images of pure cellulose (a), MCC (b), and homophthalic acid-cellulose aerogel (HPA-CA) (c) can be seen in Figure 2. It is clear from examining the figure that pure cellulose displays a customary fibrous and microfibrillar structure, comprising intricate porous networks. Furthermore, voids within the structure and a distinctly interconnected three-dimensional network can be observed. Unlike pure cellulose, MCC has a more uniform and even surface due to its higher crystal density. The porous structure of the MCC underwent a transformation into a solid and regular block configuration with defined edges. Upon reaction with HPA, MCC exhibited a unique product morphology. The resulting hybrid product, as shown in Figure 2c, displayed a heterogeneous structure with porous areas. The greater porosity of the new product in comparison to MCC can be attributed to the formation of novel regions resulting from the integration of HPA into the structure, enabled by the solvent and subsequent replacement of these regions with air during freeze-drying. In addition, SEM images of both products undeniably demonstrate a strong interaction between MCC and HPA, resulting in a completely unique material.

### 3.3 FTIR Analysis

**Figure 3:**
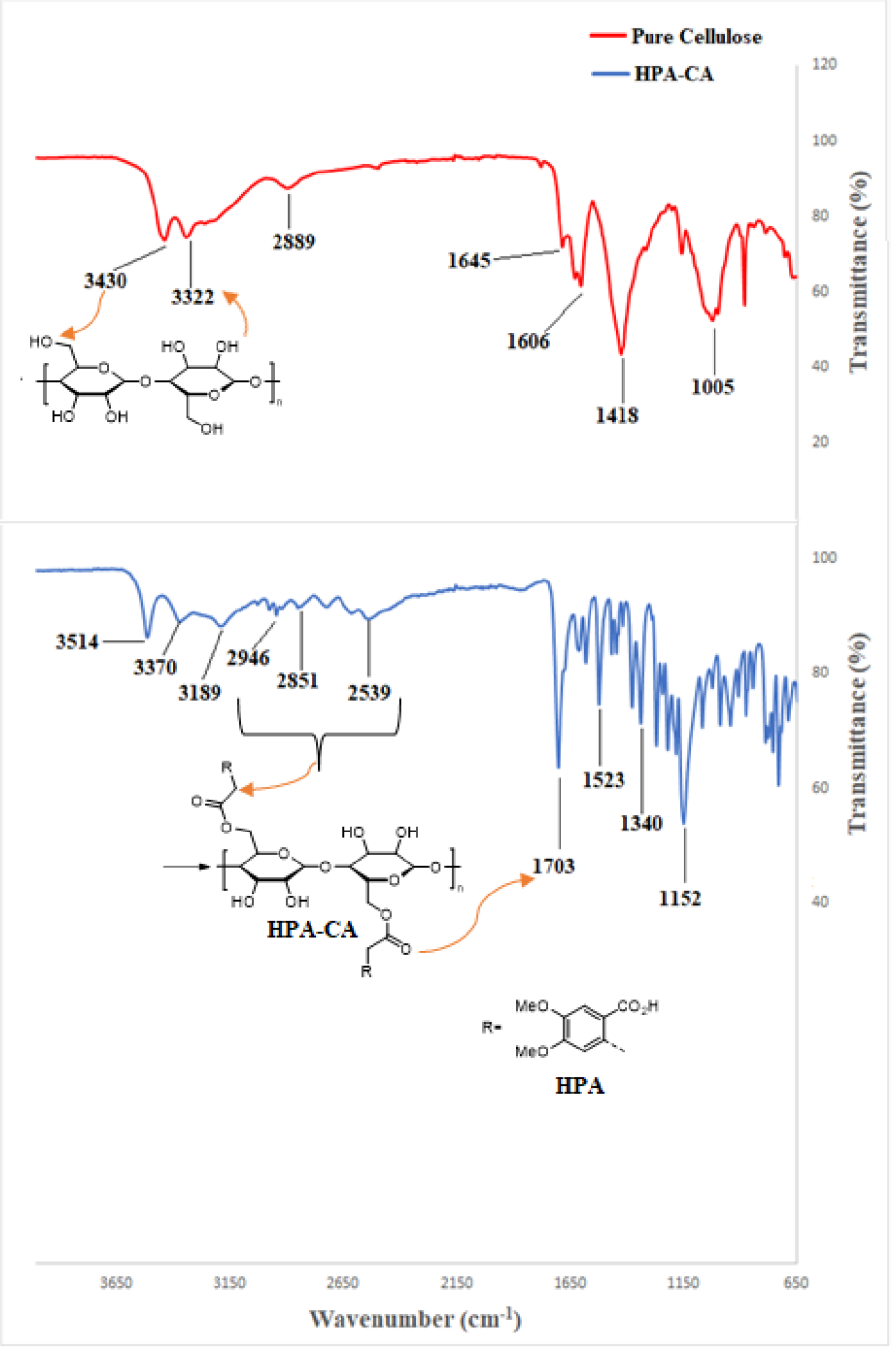
FTIR spectra of HPA-CA (blue line) and cellulose (red line).

FTIR has an important place in the structural analysis of the HPA-CA aerogel. This is because the HPA molecule cross-linked to cellulose causes serious changes in the physical properties of pure cellulose. FTIR analysis was used to observe this effect structurally in the presence of functional groups. Polyhydroxyl groups in the cellulose structure cross-link with the HPA molecule to form an ester bond. The identification of functional groups can be further supported by the FTIR spectra. The FTIR spectra of the hybrid HPA-CA aerogel sample (blue line) and pure cellulose (red line) are shown in Figure 3. If we examine the FTIR spectrum of the hybrid (HPA-CA) molecule, we can observe a relative decrease in bandwidth and density of OH groups in cellulose upon binding of HPA in the range of 3400–3550 cm^–1^.^34,35^ Furthermore, within a wide band range (2500–3000 cm^–1^), we can detect large and small O–H and C–H stretching peaks that correspond to the carboxylic acid groups of HPA and the cellulose skeleton structure. The carboxylic acid carbonyl peak (C=O), which is characteristic, can be observed at 1703 cm^–1^.^36^ This peak is not present in pure cellulose. In all samples screened, the peaks at 2851 and 2889 cm^–1^ can be attributed to the C–H stretching bands, while the peaks at around 1005 and 1152 cm^–1^ are identifiable as the characteristics of C–O ether linkages’ stretching, which may be attributed to the cellulose structure of aerogels.^37^ The bending band observed at 1418 cm^–1^ is associated with intermolecular hydrogen bonding at the CH_2_ groups within the pure cellulose sample.^38^

### 3.4 XRD Analysis

**Figure 4:**
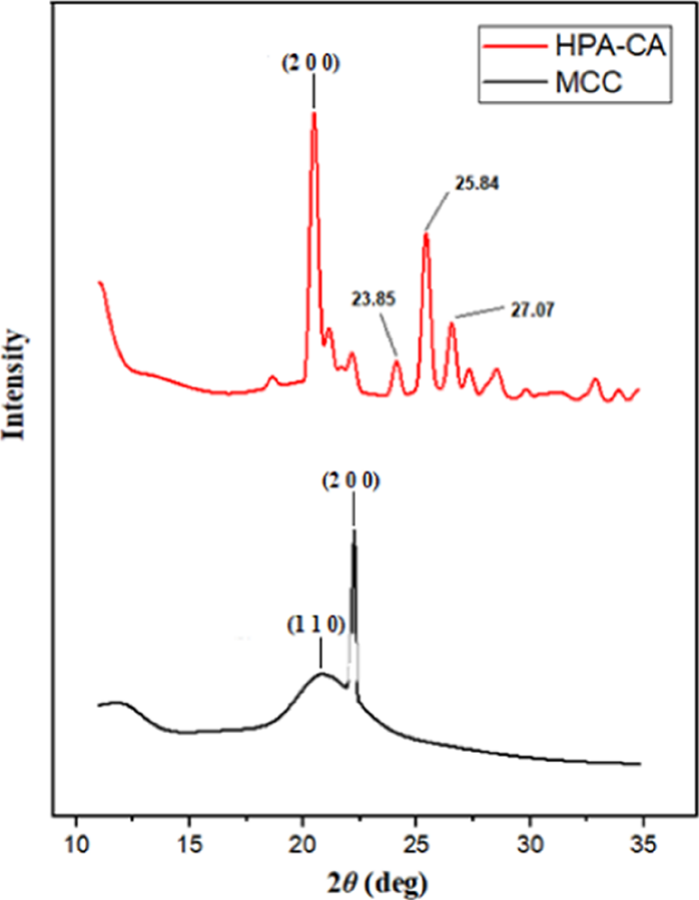
XRD patterns of HPA-CA (red line) and MCC (black line).

Cellulose is a macromolecule that contains both amorphous and crystalline regions in its structure. The crystallinity index (CrI) is a measure of the ratio between these two structural components. When examining the XRD diffractogram of the MCC sample in Figure 4 (indicated by the black line), it becomes evident that it possesses a highly crystalline structure with typical diffraction peaks for the (2 0 0) plane at 2θ = 22.21° and (1 1 0) at 2θ = 20.82°. The crystallinity index (CrI) was calculated using an empirical equation based on the intensity of the peak (I2 0 0) developed by Segal et al.^39^ (2 0 0) and the minimum intensity (*I*_am_) observed between the peaks 2 0 0 and 1 1 0:
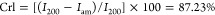
1When considering the XRD pattern for the HPA-CA sample (depicted by the red line in Figure 4), the main peak in the typical (2 0 0) plane of cellulose shifted from 22.21 to 20.61°. The crystallinity index also decreased to approximately 53% for HPA-CA. This decrease was attributed to the replacement of hydroxyl bonds by larger carboxyl bonds. Thus, it indicated the presence of large irregular areas and more amorphous forms of cellulose, as shown in XRD diffractograms. In addition, as shown in Figure 4, there are distinct peak positions at 23.85, 25.84, and 27.07° (2θ), which are not observed in the MCC structure but are indicative of the effect of the HPA compound. These peaks are thought to be crystal structures belonging to new ester components because of the interaction of cellulose, the marker molecule, with HPA. However, these structures did not contribute to the crystallinity index of cellulose.

### 3.5 Water-Absorbing Analysis

**Figure 5:**
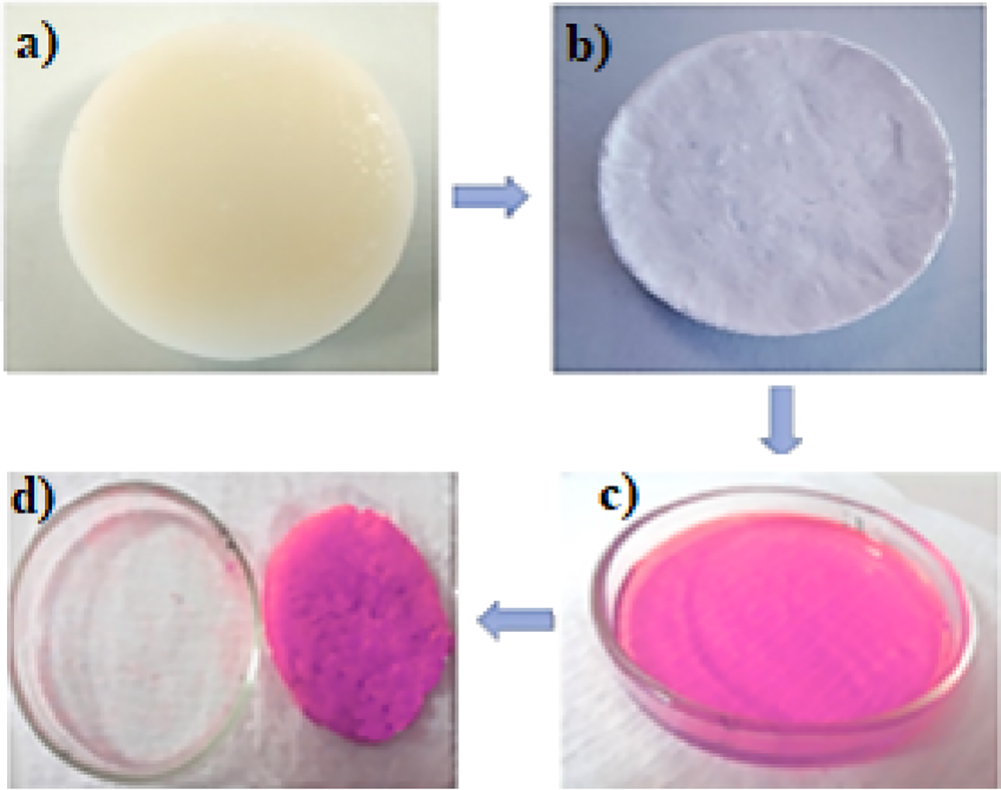
Absorbed performance of HPA-CA for dye solution ((a) solvogel, (b) aerogel, (c) Rh-B solution, and (d) Rh-B-absorbed aerogel).

The cellulose molecule is primarily composed of polar hydroxyl groups in its chemical structure, which results in a strong affinity for polar liquids like water. Additionally, the interaction of the cellulose aerogel with water has been observed to be high due to its hydrophilic components, such as −COOH groups, as seen in HPA. Cellulose aerogels produced because of cellulose esterification, such as phthalic acid, exhibit superabsorbent properties. These aerogels attract water into the material rather than store it within their structure, retaining water in the gaps between adjacent fibers. Water absorption tests have demonstrated that approximately 1 g of aerogel can absorb an average of 85 g of water. In contrast, the water absorption capacity of filter paper (Whatman) is only 1.7 g of water per gram of dry paper. The aerogel’s performance in absorbing rhodamine B (Rh-B) aqueous solution is depicted in Figure 5c. In the figure, the sample on the top left is called a sol or solvogel (a). On the top right (b), you can see the HPA-CA aerogel obtained by freeze-drying this gel. Below these, in a Petri dish containing a Rh-B solution, it can be observed that the aerogel absorbs almost the entire liquid (Figure 5d).

### 3.6 Textural Analysis

**Figure 6:**
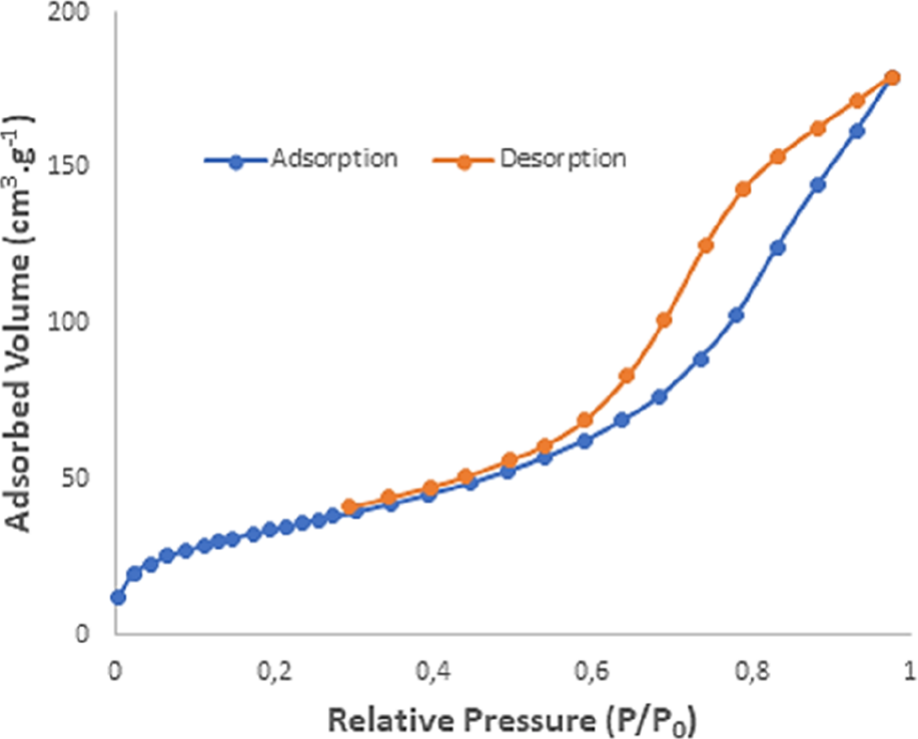
Nitrogen adsorption–desorption isotherms, HPA-CA aerogel.

Textural data concerning surface areas and internal as well as external pore structures can be acquired from the nature of nitrogen adsorption–desorption isotherms. The nitrogen adsorption–desorption isotherms of the synthesized HPA-CA aerogel exhibit characteristics resembling those of a type IV isotherm (Figure 6). This observation can be interpreted as the filling of mesopores (type IV) at higher pressures. Furthermore, according to the IUPAC classification, the distinguishing features of a type IV isotherm include a reversible portion at relatively low pressures and a hysteresis loop at relatively high pressures.^40^ The textural analysis data, obtained by applying mathematical methods to the data from adsorption–desorption curves, include specific surface areas, total pore volumes, and pore structures (such as pore type, volume, size, and area) of the sample. To analyze the specific surface area and pore structure of the aerogel samples, nitrogen adsorption and desorption isotherms were measured at 77 K and are shown in Figure 6. According to the IUPAC classification, all obtained isotherms are type IV, indicating that the aerogels are mesoporous adsorbents with strong adsorbate–adsorbent interactions. The specific surface area is influenced by porosity, and the grain size and shape play a crucial role in estimating the structural characterization of aerogels. For instance, the specific surface area was determined from the adsorption–desorption isotherms in Figure 6 using BET analysis, with values below a relative pressure of 0.3.^41^ Morphological properties and the density of the aerogel samples are provided in Table 2. The average density of the sample was found to be 0.22 g·cm^–3^. The distributions of mesopore diameters were determined from desorption isotherms using the Barrett–Joyner–Halenda (BJH) method, considering open cylindrical pores. The surface area value of the aerogel was determined to be 221 m^2^/g. As a result of the comparison of the data in Table 2 with many other cellulose-derived aerogels in the literature,^42^ it is understood that the surface area of the HPA-CA cellulose-derived aerogel has a higher value. Dye absorption data also confirm this superior feature. Undoubtedly, the contribution of HPA to cellulose is undeniable. In this study, the high liquid absorbency of HPA-CA was emphasized. Therefore, the high surface area of HPA-CA compared to many similar cellulose aerogels in the literature is valuable in terms of achieving the desired results of the study. The pore properties of the aerogel samples, including mesopore volume, total pore volume, and the percentage of mesopores, are also summarized in Table 2. The data indicate a total pore volume of 0.42 cm^3^/g for the aerogel, with a significant percentage of the mesopore volume reaching 89%.

**Table 2:** Specific BET Surface Area and Pore Structural Data of the Aerogels

**sample**	**S**_**BET**_**(m**^**2**^**·g**^**–1**^**)**	**density** (g/cm^3^)	**d**_**A**_^a^	**V**_**Total**_^b^**(cm**^**3**^ **g**^**–1**^**)**	**mesopore volume**^c^**(%)**
HPA-CA	221	0.22	7.11	0.42	89
CNC^42^	20–66	0.02–0.03			95–98.7
NCF^42^	11–15	0.0053–0.03			98.2–99.7
BC^42^	200	0.008			

a BJH average pore diameter.

b Total pore volume determined at *P*/*P*_0_ = 0.995.

c Percentage of mesopore volume.

## 4 Conclusions

Experimental data and results showed that new value-added environmentally friendly products can be produced, especially from cellulose-based waste products. By esterification of cellulose with carboxylic acids, it was revealed that this production range can be expanded within the limits of “green chemistry”. It was also revealed that it is possible to synthesize hybrid molecules that are not only limited to the production of a new and waste product but also have superior properties such as absorbency, lightness, and sustainability. In this context, the most striking results for the hybrid aerogel product are 85 g water/1 g aerogel sample for water absorbency and 0.22 g·cm^–3^ for density. In this study, an efficient, easy-to-produce, lightweight, low-cost, and environmentally friendly biopolymer material with considerable surface area has been produced. Thus, it is predicted that this product has a great potential application area in the future of materials chemistry and may be suitable for industrial-scale production.
